# Draft genome sequence of *Bacillus atrophaeus* X3, a pigmented subantarctic soil isolate from Magallanes Region, Chile

**DOI:** 10.1128/mra.00826-26

**Published:** 2026-09-11

**Authors:** Alex Gonzalez, Nancy Calisto-Ulloa, Francisco Isotta, Bárbara Seguel-Vinés, Pedro Simeone, Brenda Modak, Mick Parra, Natalia Valdés

**Affiliations:** 1 Programa de Investigación en biomedicina y biotecnología, Departamento de Salud, Universidad de Los Lagos 28061, Osorno, Chile; 2 Quimica y Farmacia, Departamento de Salud, Universidad de Los Lagos 28061, Osorno, Chile; 3 Centro Científico y Tecnológico de Excelencia Ciencia and Vida, Santiago, Chile; 4 Facultad de Ciencias, Universidad San Sebastián 373328 , Santiago, Chile; 5 Programa de Magister en Bioinformática, Universidad de Magallanes 28062, Punta Arenas, Chile; 6 Laboratorio de Productos Naturales y sus Aplicaciones, Centro de Biotecnología Acuícola, Facultad de Química y Biología, Universidad de Santiago 428356, Santiago, Chile; 7 Biomedical Engineering, Faculty of Engineering, Universidad de Santiago de Chile, Santiago, Chile; Rochester Institute of Technology, Rochester, New York, USA

**Keywords:** *Bacillus atrophaeus*, subantarctic soil, draft genome, Chile, whole-genome sequencing, cold adaptation

## Abstract

*Bacillus atrophaeus* strain X3, isolated from subantarctic soils near Laguna Amarga, Magallanes Region, Chile, harbors a high-quality genome (three contigs, 4.08 Mb, 43.4% GC content). Its genomic sequence provides insights into cold adaptation mechanisms in the *Bacillus subtilis* group, with potential relevance for emerging biotechnological applications.

## ANNOUNCEMENT

*Bacillus atrophaeus* is a spore-forming, aerobic, gram-positive bacterium with metabolic versatility and biotechnological potential in agriculture, environmental biotechnology, and industrial bioproduct production (1, 2).

Strain *B. atrophaeus* X3 was isolated in January 2025 from a soil sample (10 cm depth) collected near Laguna Amarga, Magallanes Region, Chile (50.976565°S, 72.743223°W; WGS84). This subantarctic environment features extreme conditions, including low temperatures and high UV radiation (3, 4). To recover viable isolates, the sample was plated on lysogeny broth (LB) agar.

A single colony was cultured in modified LB broth at 25°C, centrifuged, and washed. Genomic DNA was extracted using the Promega Wizard Genomic DNA Purification Kit, quantified using a Qubit dsDNA HS Assay Kit, and verified by agarose gel electrophoresis (>12,000 bp). DNA (2,500 ng) was sheared to ~9,685 bp using a Covaris g-TUBE and evaluated by Bioanalyzer. Sequencing libraries were constructed with the SMRTbell Express Template Prep Kit 2.0 and size-selected (>5 kb) on a BluePippin system, yielding 20.3 ng/µL. Sequencing was executed on the PacBio Sequel II platform using Chemistry 3.0 (30-h movie time), producing 19,401 long reads with an N50 of 9,887 bp and achieving a 68.51× genome coverage.

Long-read assembly was performed using DNASTAR Lasergene SeqMan NGen v17.6.2 (5). The final assembly yielded 3 contigs totaling 4,079,494 bp, with a contig N50 of 4,242 kb and a GC content of 43.4%. The main bacterial chromosome was assembled into a single large scaffold of 4.03 Mb with 68.51× coverage depth (representing 99.8% of the assembled reads), while two minor unlocated contigs (26.7 and 26.0 kb) accounted for the remaining sequence. Assembly quality was evaluated using QUAST v5.3.0 (6) and CheckM2 (7) with default parameters, indicating 99.93% completeness and 1.2% contamination.

Taxonomic assignment performed with GTDB-Tk v2.5.2 (8) using the GTDB release R226 (9) corroborated the identification of the strain as *B. atrophaeus*, yielding an average nucleotide identity (ANI) of 98.4% and an alignment fraction of 0.924 relative to the reference genome *B. atrophaeus* GCF_001584335.1. Closely related species (*mojavensis*, *halotolerans*, *amyloliquefaciens*, and *subtilis*) showed lower ANI values (80%–83%), confirming species-level classification.

Genome annotation performed with Bakta v1.7.0 (10) identified the following features: coding density 87.6%, 3,945 coding sequences, 24 ribosomal RNAs, 82 transfer RNAs, 2 transfer-messenger RNAs, 25 non-coding RNAs (ncRNAs), 60 ncRNA regions, 21 pseudogenes, 107 hypothetical proteins, 5 Open Reading Frames, and no assembly gaps. Two putative chromosomal origins of replication were identified, whereas origin of vegetative replication and origin of transfer elements were absent. Functional annotation identified genes for environmental adaptation. eggNOG-mapper v2 performed Clusters of Orthologous Groups (COG) classification (Table 1), emphasizing metabolic, signal transduction, and cell wall functions (11).

**TABLE 1:** Functional classification of predicted protein-coding genes from *Bacillus atrophaeus* X3 according to Clusters of Orthologous Groups (COG) categories

COG category	Gene count	% of total	Biological function/description
S	980	28.6	Function unknown
K	295	8.6	Transcription
E	260	7.6	Amino acid transport and metabolism
M	200	5.8	Cell wall/membrane/envelope biogenesis
P	195	5.7	Inorganic ion transport and metabolism
G	175	5.1	Carbohydrate transport and metabolism
J	173	5.0	Translation, ribosomal structure, and biogenesis
C	170	5.0	Energy production and conversion
L	140	4.1	Replication, recombination, and repair
H	110	3.2	Coenzyme transport and metabolism
T	105	3.1	Signal transduction mechanisms
I	90	2.6	Lipid transport and metabolism
F	88	2.6	Nucleotide transport and metabolism
O	80	2.3	Post-translational modification, protein turnover, and chaperones
V	60	1.7	Defense mechanisms
Q	48	1.4	Secondary metabolites biosynthesis, transport, and catabolism
D	45	1.3	Cell cycle control, cell division, and chromosome partitioning
EGP	35	1.0	Combined/multi-functional assignment (energy, amino acid, and inorganic ion)
U	30	0.9	Intracellular trafficking, secretion, and vesicular transport
IQ	30	0.9	Combined functional assignment (lipid metabolism and secondary metabolites)
N	30	0.9	Cell motility
Other	93	2.7	Other/not elsewhere classified
Total	3,432	100.0	

## Data Availability

The whole-genome shotgun sequencing project for *Bacillus atrophaeus* strain X3 has been deposited in the DDBJ/ENA/GenBank databases under the accession number JBUCJZ000000000. The genome assembly version analyzed in this study corresponds to JBUCJZ000000000. The associated BioProject and BioSample accession numbers are PRJNA1416488 and SAMN54969900, respectively. The sequence read data are under the accession SRX34134202.
